# The Social Networks and Social Support of Siblings of Children with Cancer

**DOI:** 10.3390/children9010113

**Published:** 2022-01-15

**Authors:** Sarah E. Wawrzynski, Melissa A. Alderfer, Whitney Kvistad, Lauri Linder, Maija Reblin, Jia-Wen Guo, Kristin G. Cloyes

**Affiliations:** 1 College of Nursing, University of Utah, Salt Lake City, UT 84112, USA; Lauri.Linder@nurs.utah.edu; 2 Intermountain Primary Children’s Hospital, Salt Lake City, UT 84113, USA; Jia-Wen.Guo@nurs.utah.edu (J.-W.G.); Kristin.Cloyes@nurs.utah.edu (K.G.C.); 3 Center for Healthcare Delivery Science, Nemours Children’s Hospital-Delaware, Wilmington, DE 19803, USA; Melissa.Alderfer@nemours.org; 4 Sidney Kimmel Medical College, Thomas Jefferson University, Woodbury, NJ 08096, USA; 5 School of Nursing, Vanderbilt University, Nashville, TN 37235, USA; whitney.kvistad@vanderbilt.edu; 6 College of Medicine, University of Vermont, Burlington, VT 05405, USA; Maija.Reblin@uvm.edu

**Keywords:** cancer, childhood cancer, adaptation, psychological, neoplasm, oncology, sibling, social support, social adjustment

## Abstract

Siblings of children with cancer need support to ameliorate the challenges they encounter; however, little is known about what types and sources of support exist for siblings. This study addresses this gap in our understanding of the social networks and sources of support for adolescents with a brother or sister who has cancer. Additionally, we describe how the support siblings receive addresses what they feel are the hardest aspects of being a sibling of a child with cancer. During semi-structured interviews, siblings (ages 12–17) constructed ecomaps describing their support networks. Data were coded for support type (emotional, instrumental, informational, validation, companionship) and support provider (e.g., mother, teacher, friend). Network characteristics and patterns of support were explored. Support network size ranged from 3 to 10 individuals (M = 6 ± 1.9); siblings most frequently reported mothers as sources of support (n = 22, 91.7%), followed by fathers (n = 19, 79.2%), close friends (n = 19, 79.2%) and siblings (with or without cancer) (n = 17, 70.8%). Friends and brothers or sisters most often provided validation and companionship while instrumental and informational supports came from parents. This study provides foundational knowledge about siblings’ support networks, which can be utilized to design interventions that improve support for siblings of children with cancer.

## 1. Introduction

A pediatric cancer diagnosis causes disruptions within the family including shifting of roles, finances, and resources. The focus on the needs of the diagnosed child often leaves siblings feeling anxious, alone, and distracted [1,2]. Siblings may experience poor psychosocial adjustment, including poor school functioning, cancer-related traumatic stress, and poorer quality of life [3,4,5]. Due to distress and challenges with adjustment among siblings of children with cancer [4,6], supportive care, including providing education and psychological supports, for siblings is recommended as a standard of care in pediatric oncology [7]. 

Social support is broadly defined as the provision of assistance, comfort, or resources to individuals that alleviate stress and assist in coping [8]. The stress-buffering hypothesis of social support [9] has been extensively explored and suggests that social support offers resources and promotes coping to buffer stress. Social support is well established as a key factor in health outcomes and adjustment in children and adolescents [10,11,12,13]. 

Few studies have examined siblings’ perspectives of their social support or associations between support and adjustment [14,15]. A recent scoping review suggested that social support is indeed helpful to siblings; however, the most important sources and types of helpful support for siblings of children with cancer remain unclear [16].

The purpose of this study was twofold. First, we aimed to advance our understanding of social support among siblings by identifying sources and types of support within their social networks. Second, we aimed to identify how support sources and types of support given to siblings alleviates the “hardest things” they have encountered since the cancer diagnosis of their brother or sister through their own narratives. 

## 2. Materials and Methods

### 2.1. Participants

All study procedures were approved by the Institutional Review Board at the University of Utah (protocol #00124303). Eligible participants were healthy adolescent siblings (age 12–17) of children in active treatment for cancer or off treatment but diagnosed within the last two years. Our sampling was purposive to ensure representation of varied ages and genders of participants because developmental age and gender are known to influence perceptions of support [10,17]. Siblings were English-speaking, nonbereaved, and living in the home of the child with cancer at least 50% of the time. Table 1 provides participant, family, and cancer diagnosis demographics.

### 2.2. Screening and Recruitment

Two methods were used to recruit participants for this study. First, we used the electronic health records (EHR) at Primary Children’s Hospital, a quaternary pediatric oncology center serving the Intermountain West of the United States, to identify pediatric patients diagnosed with cancer within the last two years. Second, we partnered with SuperSibs, a program of Alex’s Lemonade Stand Foundation, to identify families with eligible siblings. SuperSibs is a free program providing comfort and care mailings to siblings of children with cancer. Parents identified through both sources received emails inviting participation in a study of sibling social networks and support. They were then contacted by phone to answer any questions and determine interest in participation. Interested caregivers completed a screening survey to confirm sibling eligibility. If eligible, parental permission and demographic information were collected via a Research Electronic Data Capture (REDCap) [18] link, along with contact information for the target sibling. Eligible siblings were then sent study information and invited to participate. Interviews were conducted after documentation of assent.

### 2.3. Data Collection

Participants completed audio-recorded interviews that lasted 20–47 (*M* = 30) minutes and took place via Zoom [19]. Participants worked with the researcher to build an ecomap [20], a visual representation of their social network, by identifying up to 10 people they perceived as a source of support throughout their brother or sister’s cancer experience. Participants described characteristics of their social support networks (e.g., relationship, age, and closeness) and interactions with members (e.g., frequency, type of support) to complete the ecomap (see Appendix A, Table A1). Participants were asked about the kind of support each person provided to them using a set of terms and examples formulated in lay language developed and tested in previous work [21]. However, they were also allowed to freely describe specific or recent examples of the support received from each source they mentioned in their ecomap without using the terms provided. 

Participants were also asked to describe what they felt was the “hardest thing” for them since their brother or sister’s diagnosis along with any support they felt helped in dealing with or alleviating stress related to their identified “hardest thing”. 

### 2.4. Data Integration and Analysis

Demographic and ecomap data were summarized using descriptive statistics (mean, percentage) which were generated using SPSS version 26 [22]. Audio recordings of participant interviews were transcribed verbatim. Transcripts were then de-identified and imported into Dedoose [23] for management and coding. Participant demographic data were imported as case descriptors and linked with interview data. 

To ensure theoretical and empirical coverage of the data, analysis took place in two stages of deductive and inductive coding. First, we used a deductive approach applying codes and definitions derived from theoretical constructs, interview questions, and review of the ecomap data (e.g., relationship and interaction characteristics, types of perceived support) [24]. This preliminary deductive coding scheme was refined by four members of our research team (SEW, WK, MA and KGC) by coding two interviews together. Primary coders (SEW and WK) then independently coded a series of interviews, discussing inconsistencies in coding and refining use of the code book after each one until reliability was established by achieving a Kappa within the “substantial” range [25], at 0.86 after four transcripts. Coders then independently coded the rest of the transcripts resolving discrepancies at weekly meetings.

Next, an inductive open coding approach with thematic analysis was undertaken to ensure novel content was captured and integrated into the coding [26]. Throughout the coding process, reflexive and analytic memos were recorded directly into Dedoose alongside the data to enhance the description and understanding of the data [27,28]. In the second phase of coding, the authors reviewed and discussed the codes and memos to develop themes and summarize the data. Codes and subcodes were organized by conceptual similarity, subsuming the initial codes within emergent thematic categories.

## 3. Results

### 3.1. Sample Demographics

Twenty-four siblings between the ages of 12 and 17 were included in this study (Table 1). Half were male and half were female. Most siblings were white, but nearly one-third represented racial or ethnic minorities. All siblings came from traditional (mother, father, siblings) or blended (divorced and remarried or cohabitating adults and their children) two-parent homes; however, families varied regarding income, parental education, cancer diagnosis, and time since diagnosis.

### 3.2. Sources of Support

**Figure 1:**
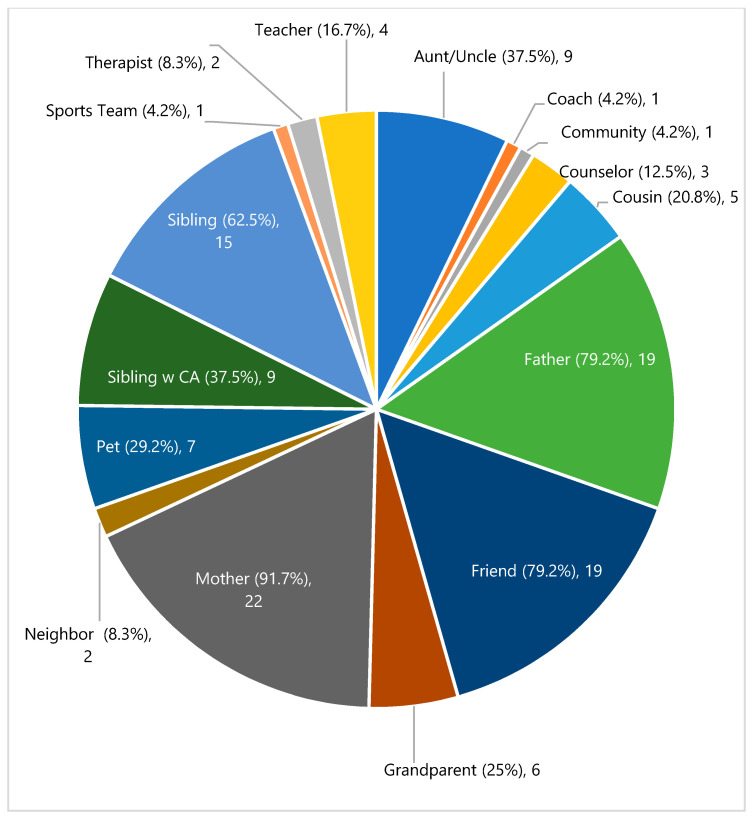
Percentage and number of siblings reporting each mentioned source of support.

Ecomaps indicated that siblings’ cancer social support networks ranged from 3 to 10 individuals. A total of 162 individuals were identified as sources of support in our sample’s ecomaps, with each individual providing one to three types of support. Siblings reported an average closeness rating of each supporter at 4.5 (SD 0.78, range 1–5) with 5 indicating the greatest perceived closeness to the individual. On average, siblings’ social networks were primarily made up of family members (71.6%, range 33–100%). Figure 1 shows the type of relationships included in each sibling’s ecomap network by percent. Mothers were identified as a source of support by nearly all participants. The next most frequently mentioned sources of support were close friends and fathers, then a brother or sister in the home. One-third of siblings (33.3%) mentioned a teacher, school counselor, or coach, and 29.1% included their family pet in their support network. Several siblings mentioned a group of individuals as a single source of support in their ecomap, for example, a sports team, their local community, or a neighboring family who offered them important supports throughout the cancer journey (Figure 1).

### 3.3. Types of Social Support Received

Siblings identified support within all six deductively derived domains, including emotional, informational, instrumental, companionship, and validation support. Two additional types of support, appraisal support and indirect support, were identified via inductive coding. In total, N = 383 examples of support were identified across all interviews; what follows is a summary of the specific supports reported by siblings within each domain of support. Percent of support provided their most frequent sources are also included. Definitions for each type of support and exemplary quotes are noted in Table 2.

Emotional support was the most frequently identified type of support siblings reported (N = 144/383, 37.6%) receiving from their social network members. Examples of emotional support among siblings often related to encouragement and “check-ins” where the identified source of support would ask how the sibling was doing or make themselves available to the sibling to talk or answer questions. Some siblings had difficulty identifying a specific example of how emotional support had been given but articulated instead that the source was “just there for them”, giving them a sense of presence and availability. Emotional support was provided across all types of sources of support; however, data matrices showed that emotional support was most frequently provided by friends (n = 35, 24.3%), mothers (n = 25, 17.4%), healthy siblings (n = 18, 12.5%), and fathers (n = 17, 11.8%).

Companionship was the second most frequently reported type of support among siblings (N = 80/383, 20.9%). Companionship primarily served the purpose of distraction for siblings. Companionship allowed siblings to feel normal, have fun and escape the cancer experience. This was in the form of sports activities, “hanging out” with friends, or a one-on-one trip to the store with a parent or older sibling. While parents and other sources of support were identified as providing companionship, friends (n = 22, 27.5%) and healthy siblings (n = 20, 25%) were the most frequently identified sources of companionship. 

Siblings also identified instrumental support (N = 53/383, 13.8%) in a variety of forms. Several siblings mentioned receiving meals or having a place to stay while their parent(s) were at the hospital. Sometimes instrumental support was help with homework, sports, or facilitating sibling participation in their interests or activities. Instrumental support was most frequently provided by fathers (n = 14, 26.4%), followed by mothers (n = 11, 20.8%) and aunts (n = 8, 15.1%). Instrumental support provided by parents also included acting as a link to other sources or types of support. For example, parents were able to connect siblings to teachers, therapists, or extended family in the sibling’s social network who provided support.

Informational support was the next most identified support (N = 50/383, 13.1%). Informational support most often related to someone providing information about cancer, its treatment, or the effects of treatment. Several siblings mentioned someone providing information that supported them in dealing with specific challenges such as tips on interacting with the child with cancer or help with homework. Informational support was most frequently identified as being provided by mothers (n = 16, 32%), fathers (n = 13, 26%), and healthy siblings (n = 8, 16%). Teachers and aunts were also mentioned by some.

Validation was identified as having someone who understands you or your experience; this was typically related to the cancer experience (N = 32/383, 8.4%). Validation was most frequently provided by healthy siblings (n = 8, 25%) and friends (n = 7, 21.9%).

We also noted two additional types of support described by participants that did not fit our initial coding approach. First, appraisal support was identified, comprised of comments or behaviors from individuals in the siblings’ network that assisted in the sibling’s self-evaluation or their appraisal of their social situation. Appraisal support (N = 15/383, 3.9%) was occasionally related to cancer, but most often related to feedback or affirmation given to the sibling unrelated to cancer, such as praise for an accomplishment or help with typical adolescent interpersonal issues. Appraisal support was provided by fathers, healthy siblings, and the children with cancer with the same frequency (n = 3, 20%) followed similarly by mothers and friends (n = 2, 13.2%). 

Second, indirect support occurring at the family level was identified (N = 9/383, 2.3%). This type of support was not directed at the sibling specifically but provided support targeted at alleviating family stressors, which provided siblings with the added benefit of feeling more secure in their situation. Examples of this included community fundraisers, GoFundMe campaigns, care of their family needs, or the care their brother or sister was receiving for their cancer. Siblings who identified these supports conveyed that these types of support helped them feel loved, watched over, or provided a sense of comfort regarding their worries about their brother or sister with cancer. Indirect support was identified as coming from medical professionals (n = 3, 33.3%), fathers (n = 2, 22.2%), and community (n = 2, 22.2%) most often. Frequencies of reported support by gender are noted in Table 3. Chi-square analysis on gender differences in reported support was significant, (X^2^ (df = 6) = 15.21, *p* = 0.019; Cramer’s V = 0.20) and appraisal support was noted to be the type of support contributing significance.

### 3.4. Hardest Things and Most Helpful Supports Reported by Siblings

Sibling reports of the “hardest thing” they had dealt with related to their brother’s or sister’s cancer aligned with their reports of the most helpful supports and the inductive themes identified in our analysis. In Table 4, we provide examples of what siblings identified as the hardest things, their most helpful supports, along with the overarching themes noted in the data. Specifically, we identified involvement in family and care, distraction, creating connections and presence, and understanding as most relevant in addressing their identified challenges. 

Seeing their brother or sister sick or their parent’s distress was most frequently reported as the “hardest thing”. Siblings reported struggling with being treated differently by parents, friends, or others. Siblings felt more alone and limited in their interactions with friends or normal routines and their social networks provided a sense of security, integration, and normalcy that was important to the siblings in coping with their identified challenges. 

Siblings identified their own ability to provide support to their family as important. Siblings took on caregiving activities out of a seeming desire to be a part of the family through the cancer trajectory. Siblings discussed the importance of spending time with their brother or sister with cancer, seeing improvements in their health, or even their joy however brief. Siblings also wanted to be a support to parents. One sibling even mentioned seeking to understand their mothers’ challenges through an aunt in their social network. Overall, siblings conveyed that assisting with the care of other healthy siblings or their brother or sister with cancer provided siblings with a sense of integration, purpose, and visibility within their family.

Distraction, often occurring through companionship, allowed siblings to feel close to their family and gain a sense of normalcy while dealing with the distress and changes caused by cancer. Concurrently, instrumental support, such as transportation or money, was important to provide the means for siblings to spend time with friends and engage in extracurricular activities, which further supported distraction from cancer.

Most siblings reported that members of their social network were aware of their challenges and were available to them. This created meaningful connections and a presence felt by siblings. Checking in and knowing people were watching out for them were often expressed as important emotional supports. Even when discussing other forms of support, the emotional significance of the time or support an individual provided was simultaneously expressed by siblings.

Finally, siblings expressed understanding as important. This occurred in two ways. First, siblings wanted “real” information. Siblings could see the distress of their parents, siblings, and others. Information that helped siblings grasp the situation, gain perspective, and feel grounded and was identified as one of the most important supports in dealing with their brother or sister’s cancer. Second, siblings felt understanding related to their feelings and challenges helped them feel seen. This type of understanding was identified in things like having extra time to do a homework assignment and leeway in their emotions and processing of the situation. 

## 4. Discussion

Previous work has identified that siblings of children with cancer are at risk for poor adaptation, difficulties in school, and altered relationships with members of their social networks [4,6]. Barriers to supporting siblings have also been identified [29,30], and structured support may not be available to many siblings. COVID-19 has further limited access to supportive services [31]. Notably, no siblings in our study mentioned receiving support or information from the oncology team or hospital for themselves, and when formal services from a therapist were obtained for siblings, it was reported as having occurred as a result of a parent or teacher concern and support. 

In this study, we aimed to advance our understanding of social support among siblings and to fill a gap in the literature by characterizing sibling social support networks and identifying the sources and types of support they find helpful. We identified that existing, informal supports were most meaningful and helpful to siblings during their brother or sisters’ cancer and that this support most often came from sources closest to them. Siblings identified specific examples of support across a variety of social support domains. The examples of support received from their networks were relatively typical for adolescents [32,33], contributed to the siblings’ sense of security, and made them feel cared for during the stressful experience of cancer within the family. These findings are consistent with other research demonstrating siblings challenges [15] and their desire to be seen and involved when a brother or sister has cancer [1,2].

In this study, we also aimed to identify through narratives how the sources and types of support given to siblings alleviate the “hardest things” they have encountered since their brother or sister’s cancer diagnosis. Sibling social networks were primarily made up of family members and close friends, highlighting the importance of support within close relationships. Unfortunately, lack of awareness of sibling support needs within the family is a recognized barrier to sibling support [30]. Based on our findings, siblings seem to benefit from meaningful connections formed when others regularly check in on their well-being and allow them space to express their specific needs. In turn, as others learn of siblings’ needs, they may be better able to provide congruent support or seek out appropriate professional support when needed. Supports such as providing distraction activities, humor, and understanding of their experience help siblings in small ways to meet the challenges they face being a sibling of a child with cancer. In addition, while most support came from parents, others such as extended family or community can support siblings (and parents) by providing these types of support.

Siblings in our study expressed being acutely aware of the challenges that their diagnosed brother or sister and parents faced and the implications of this on themselves. Siblings also indicated that their parent and family members’ well-being was important and contributed to their own sense of emotional security and coping. Previous research has documented similar findings noting that pre-existing family challenges, inadequate resources, or poor parental coping can contribute to poor adjustment to cancer in all family members including siblings [34,35]. Clinicians can routinely assess for these psychosocial issues within families, stratify risk, and improve health equity using tools like the Psychosocial Assessment Tool [36].

Important sibling supports were often related to being seen, involved, or part of the family. Our overarching theme of being “involved” may be more about siblings leveraging their own power to create or enhance cohesion and connection between themselves and their important supporters, rather than a desire for increased responsibility at home or in the care of the child with cancer. These findings align with family systems theory [37] and suggest that family focused interventions may be the most impactful for siblings because positive changes within their relationships with their most important social network members—family members—may enhance intervention effects. 

Clinicians treating children with cancer can use our findings to offer additional evidence and guidance to parents about keeping adolescent siblings involved, supported, and connected as they navigate the cancer trajectory. Our findings point to helpful support coming from siblings’ existing and informal social networks, available to siblings in their day-to-day activities, outside of structured hospital and community-based interventions. Previous research has demonstrated that siblings of children with other chronic illness experience similar emotional and psychological challenges [38,39] to those of children with cancer. Our reported findings should be compared to those reported by siblings of other childhood illnesses and may be applicable and useful in supporting other sibling groups. 

This is among the first studies to report on the social networks of siblings, and our findings should be interpreted with caution. While participant selection was purposive, the sample was relatively small and under-represents the racial and ethnic diversity that is prevalent in the general population of adolescents in the United States [40]. In addition, many children who participated were recruited from SuperSibs, a program that recognizes the needs of siblings of children with cancer. These families may be more aware and in tune with sibling support needs. Finally, our sample was entirely composed of two parent families; the known challenges for single parent families [41] were not integrated into our findings. Our findings may in fact represent a “best case”, as participants often expressed having adequate supportive resources. 

It is important to note that siblings with supportive resources may still have unmet social support needs if the support they receive is mismatched to their specific challenges. These mismatches of support and need may play a role in poor or ineffective adjustment to the cancer experience. Furthermore, while emotional support was the most frequently reported type of support, it may not be the most needed; rather it may be the most easily offered or cognitively accessible to this age group. More work is needed to determine the specific support needs of individual siblings and how to leverage the supports available to them to promote their healthy adjustment. Future studies could undertake a more traditional social network analysis examining how support, cohesion, or the heterogeneity of their network influences sibling outcomes. Lastly, our research noted some differences in reports of appraisal support by gender, and other research suggests that cultural influences play a role in what supports are desired [42]. Additionally, this generation is the most diverse generation in US history [40] (race, ethnicity, orientation and gender identity), and that should be accounted for in research. Future research efforts should further examine and confirm if specific types of support are more relevant to specific demographic groups or socioeconomic aspects of families.

## Figures and Tables

**Table 1:** Participant Demographics.

	Range	Mean (SD)	N	(%)
Age	12–17	14.2 (1.6)		
Gender	Female		12	50
	Male		12	50
Race	Asian		1	4.2
	Black		3	12.5
	White		20	83.3
Ethnicity	Hispanic or Latinx		6	25
Family Situation	Traditional		21	87.5
	Blended		3	12.5
Family Income ^1^	Less than 500,000		6	25
	50,000–99,000		3	12.5
	150,000–199,999		7	29.2
	200,000–249,999		3	12.5
	250,000–299,999		2	8.3
	More than 300,000		1	4.2
Time since	0–3 months		2	8.3
Diagnosis	3–6 months		5	20.8
	6–12 months		5	20.8
	12–18 months		3	12.5
	18–24 months		4	16.7
	Over 2 years		5	20.8
Diagnosis	Leukemia		11	45.8
	Lymphoma		6	25
	Sarcoma		4	16.7
	Solid Organ		2	8.3
	Brain		1	4.2
Parent Education	Some College		5	20.8
	Vocational or Specialized Training		3	12.5
	Bachelor’s Degree		11	45.8
	Master’s Degree		4	16.7
	Doctoral Degree		1	4.2

^1^ Two families did not wish to disclose income.

**Table 2:** Social support examples from siblings by category of support.

Social Support	Codebook Definition	Example Quote
Emotional	Receive empathy, caring, reassurance, or encouragement. Knowing you have someone available who cares about you.	“She would ask questions about my feelings and stuff. Because, as a sibling,—it feels bad to think that you’re going through something, rather than your sibling’s going through something. So, like it was easy to talk to her.” (16 y/F, talking about a cousin)
Informational	Receiving knowledge, recommendations, or advice.	“He was kind of like—it’s going to be okay. He was the one—more than my mom, he was the one who kind of gave me the info on his cancer and he kind of like informed me what was going on and he did it in a nice way and everything.” (15 y/F, talking about her dad)
Instrumental	Receipt of services such as transportation, money, or help with household chores, homework, and skill building.	“She helped me by cooking for me and providing me meals.” (13 y/M, talking about his grandmother) “yeah, and still helping me with homework even though he’s busy.” (12 y/M, talking about his dad)
Companionship	Spending time together for distraction an escape from cancer, offers reprieve and fun.	“I would say that he just like—he’s so tiny that he doesn’t really understand it fully yet. So, he just helps me just get my mind off of it and just like “Hey, [brother], you want to play Legos or something?” I’m like “Okay, sure.” (14 y/M, talking about younger sibling)
Validation	A sense of belonging and shared world view, having someone who understands you.	“She understands, and she needs people to talk to just as much as I do” (12 y/F, talking about a sibling) “He has a relative, I think, who had cancer, and it’s a different kind of cancer, of course, but he tells me all the time that he knows how hard it is, and he’s there to help.” (16 y/F, talking about a friend)
Appraisal	The provision of affirmation, or feedback for self-evaluation and social comparison.	“[brother w CA] was telling me about how for the past two years he was depressed because he was in and out of hospitals. He couldn’t get to see us, and that really inspired me to try my best for him.” (16 y/M talking about his older brother) “I was giving a speech to my prayer center, my mosque. And it went pretty well, and my dad gave me some feedback and told me how I could improve, what I did well, and he did it all in a very nice, friendly way.” (14 y/M talking about his dad)
Indirect	Supports siblings report has helpful to them but are not directed at them specifically.	“People in my ward, they would bring us dinner,—they mowed our lawn, and they are constantly visiting us, trying to help with anything…it helps me feel like people care, and we have help if we need it, and that’s comforting.” (16 y/F talking about her neighbors)

**Table 3:** Frequency of Social Supports Reported by Gender.

Social Support	Gender	
	Male	Female
Emotional	61	83
Informational	20	30
Instrumental	24	29
Companionship	30	50
Validation	7	25
Appraisal	12	3
Indirect	4	5

**Table 4:** Hardest and most helpful supports reported by siblings.

Hardest Things Since CA Dx	Overarching Themes	Most Helpful Supports
“When [brother w CA] is not feeling good, or when he starts feeling sick, during the chemo, or he’s weak, and he’s crying, like that’s the hardest thing, because I don’t like to see him in pain.” (P22) “Probably mostly just feeling bad for him, like just all the hard things that he’s had to go through.” (P19) “Just accepting that things won’t be the same for [sister w CA]…she is super tired and we can’t joke around and she is getting super serious.” (P14) “I think that’s the hardest thing is just seeing my mom not take care of herself, and I think the hardest thing is just thinking into the future.” (P1)	Involvement in Care and Family	“I try to like comfort him. Because usually, when he’s like feeling like that, he’ll ask for me. I’ll come and just lay with him, watch a movie with him, and just try to comfort him as best as I can.” (P22) “I think the thing that has helped me the most is that [my siblings] understand that like she’s sick and stuff, and they’ll help me make cards for her and things.” (P11) “Just hanging out with [sister w CA] more maybe.” (P14)
“A lot of the time my dad would be working and mom would take him to the hospital, so I’ll be by myself.” (P15) “I think probably feeling more alone because I was probably closer with my parents before my brother got diagnosed and obviously, my brother was in the hospital like a bunch of different times.” (P11) “I would say probably the attention, like less attention.” (P6)	Creating Connection and Presence	“When everybody’s together.” (P24) “[Aunt], she is just be there for us, to check up on us when we were down, and she was just there.” (P22) “Well, it’s always nice to like see people—see that people care and want to help you.” (P19) “Probably just them being open to talk, being like “Hey, if you want to hang-out we can hang-out.” (P15)
“Selfishly, the hardest thing has been just my mental health getting really bad since then. It kind of just downward spiraled since he was diagnosed.” (P5) “Pretty much not being able to see people a lot and go places.” (P12) “Losing friends” (P21) “Dad would be working, and my mom will have to take [brother w CA] to the hospital, and so I’ll be by myself for a few weeks just at the house.” (P15)	Distraction	“I’d just say like going to practice gets my mind off it, like my dad taking me to practice. I don’t really think about it while I’m there.” (P23) “I really like hanging out with my cousins and with some of my friends online.” (P16) “You know, I could still do my music lessons…my theater classes. I had people to drive me to those. And I could do a show or something, because of that support that I had. Kept me feeling like, “Okay, my life is still going to go on. This just happened to my brother, but I can still live my life and do my things.” (P5)
“When by brother first got diagnosed they were open to me asking questions…but I guess they got tired of it” (P21) “When I didn’t know anything about it, I wasn’t sure if he was going to be okay.” (P17) “Well, I feel like if someone finds out that [my sister] has cancer, they’ll be like, “Oh, I’m so sorry”, and like feel all awkward if I tell them that like, “It’s really not a big deal”, and so I feel like that’s hard and I never really understood it all the way.” (P11) The hardest thing has probably been schoolwork … a result of distractions and stuff like that. You just don’t know what’s going to happen next, your mind is in a million different places. (P4)	Understanding	“I think the most helpful was knowing what was going on with my brother. I appreciate my mom the most for telling me straight-up what was going on with my brother. I felt like that kept me grounded the most.” (P21) “I don’t know, [my parents] gave me time and space and just like processing room. So, I feel like homework, I could have an extended amount of time or something or like with like different things, they’d be like “Oh, yeah, I understand.” (P10) “Probably just like having a few people that understand.” (P13)

## Data Availability

The data presented in this study are available upon reasonable request from the corresponding author. The data are not publicly available due because participants of this study did not agree for their data to be shared publicly.

## Appendix A

**Table A1:** Interview Guide.

ECO-MAPPING INTERVIEW GUIDE
In this interview we are going to talk about the people in your life that provide you with support and help you. First, think about the people in your life who have supported you. This can be people that you see in person every day, or people that you text, talk or chat with online. We’re going to make a list of people you feel have supported you the most throughout your sibling’s cancer diagnosis and treatments. Together, we are going to make a diagram of your relationships with these people. Each circle will represent a person that you feel supports you. We are going to put your name in the center circle. Next let’s add the names of the people and relationships you have been thinking about. We are going to talk about each person on your map here. I’m going to ask you a few questions about each person, including their relationship with you, and how they support you. As we go, you can make changes to the map, by adding or subtracting people from your map. *For each person identified in the siblings eco-map ask the following questions:* What kinds of support does this person give you? Can you give me a recent example of support that this person or interaction provided you? On a scale from 1–5 how close do you feel to this person? (1 being not very close, an acquaintance that supports you and 5 being someone very close that you feel you could reach out to at any time for support) Do you see or talk with this individual in-person? How often? (Multiple times a day, once a day, few times a week, few times a month) Do you see or talk with this individual online or over the phone (texting, phone call, skype)? How often? (Multiple times a day, once a day, few times a week, few times a month) Now that you’ve thought about the people and support you have received, I am going to ask you a little bit more about your experiences as a sibling of someone with cancer. 6. What types of support do you think are the most helpful to you? Please describe how or why? 7. Do you ever use social media or the internet to get support? For example, to find information about cancer or to find other siblings like you who may be going through the same thing? 8. Does using social media [e.g., Instagram, YouTube, Snapchat] help you get the support you need? If yes, can you give me an example? 9. Do you think your SM or technology use has changed since your sibling was diagnosed with cancer? If so, how? 10. Can you talk about how confident you are in your ability to make friends online or seek out the help/support when you need it? 11. What has been the hardest thing for you since your [brother or sister] got sick with cancer? 12. Has there been anything in particular that you think has helped you deal with that? 13. *[Referring to their Ecomap]* How do you think these connections have changed since your sibling was diagnosed with cancer? [Alternative prompt: Do you think you would have made a different map before your [brother or sister] got cancer?] 14. How do you think things have changed or are different regarding your social support and connections with others since the COVID pandemic? Remember when schools closed, how have things changed with your friends or other people in your network here? Can you think of anything else that you think is important for us to know about your support system or what might be helpful to other siblings like you?
